# A Differential Drug Screen for Compounds That Select Against Antibiotic Resistance

**DOI:** 10.1371/journal.pone.0015179

**Published:** 2010-12-08

**Authors:** Remy Chait, Shreya Shrestha, Aakash Kaushik Shah, Jean-Baptiste Michel, Roy Kishony

**Affiliations:** 1 Department of Systems Biology, Harvard Medical School, Boston, Massachusetts, United States of America; 2 Faculty of Arts and Sciences Center for Systems Biology, Harvard University, Cambridge, Massachusetts, United States of America; 3 School of Engineering and Applied Sciences, Harvard University, Cambridge, Massachusetts, United States of America; Columbia University, United States of America

## Abstract

Antibiotics increase the frequency of resistant bacteria by providing them a competitive advantage over sensitive strains. Here, we develop a versatile assay for differential chemical inhibition of competing microbial strains, and use it to identify compounds that preferentially inhibit tetracycline-resistant relative to sensitive bacteria, thus “inverting” selection for resistance. Our assay distinguishes compounds selecting directly against specific resistance mechanisms and compounds whose selection against resistance is based on their physiological interaction with tetracycline and is more general with respect to resistance mechanism. A pilot screen indicates that both types of selection-inverting compounds are secreted by soil microbes, suggesting that nature has evolved a repertoire of chemicals that counteracts antibiotic resistance. Finally, we show that our assay can more generally permit simple, direct screening for drugs based on their differential activity against different strains or targets.

## Introduction

Treatments with antibiotics are intrinsically selective processes. While antibiotics are primarily chosen based on their absolute effect on target pathogens, they also strongly affect the relative competitive advantage of drug resistant versus sensitive strains. As a result, use of an antibiotic promotes the emergence and spread of strains resistant to it. In the face of a decline in discovery of new antibacterials, the rise of these resistant strains has led to an acute emerging health concern [1].

While an antibiotic confers a strong selective advantage on bacteria resistant to it, certain compounds exist that can reduce or even invert this advantage. For example, compounds which disable resistance mechanisms, such as inhibitors of drug-degrading enzymes or of efflux pumps can neutralize the advantage of resistance [2], [3]. Other compounds can go even further by completely inverting this selective advantage, causing the resistant bacteria to lose in competition with their sensitive cousins. Such selection-inverting compounds can be classified into two types. First, selection against a specific resistance mechanism can occur when this mechanism also operates to enhance the toxicity of certain compounds [4], [5] (e.g., tetracycline efflux pumps increase sensitivity of *Escherichia coli* to fusaric acid) [6]. Such mechanism-mediated selection against resistance is often termed ‘negative cross-resistance’ or ‘collateral sensitivity’ [7]. Second, we have recently shown that inversion of selection can occur when the antibiotic to which the bacteria are resistant is combined with a toxin whose potency is suppressed by the antibiotic [8], [9]. Since resistance effectively eliminates the antibiotic, it removes not only the particular inhibitory effect of the antibiotic, but also removes the protection from the toxin provided by the antibiotic. Where the latter effect is greater, the result is a net reduction in the fitness of the resistant relative to the sensitive strain (e.g., inhibition by ciprofloxacin is suppressed by tetracycline and their combination can select against tetracycline resistance) [8]. Since this second class of selection-inverting compounds do not directly select against resistance genes, but instead select on the physiological consequences of the reduced antibiotic effect that the genes bring about, we say that the resulting selection is “potentiated” by the resisted antibiotic. Since selection inversion by the first class is mediated by the resistance genes alone, without requiring the presence of the drug to which the bacteria are resistant, we refer to it as “direct”.

Motivated by the examples of compounds known to exert direct or potentiated selection against resistance, we asked how common such compounds are and devised a general strategy to screen for them. By combining competition between fluorescently labeled bacterial strains with a robust test of bacterial drug susceptibility, we have produced a simple and flexible assay which allows visualization of both the absolute inhibitory effect of compounds as well as the differential selection that they impose on strains sensitive and resistant to a chosen antibiotic.

## Results

**Figure 1:**
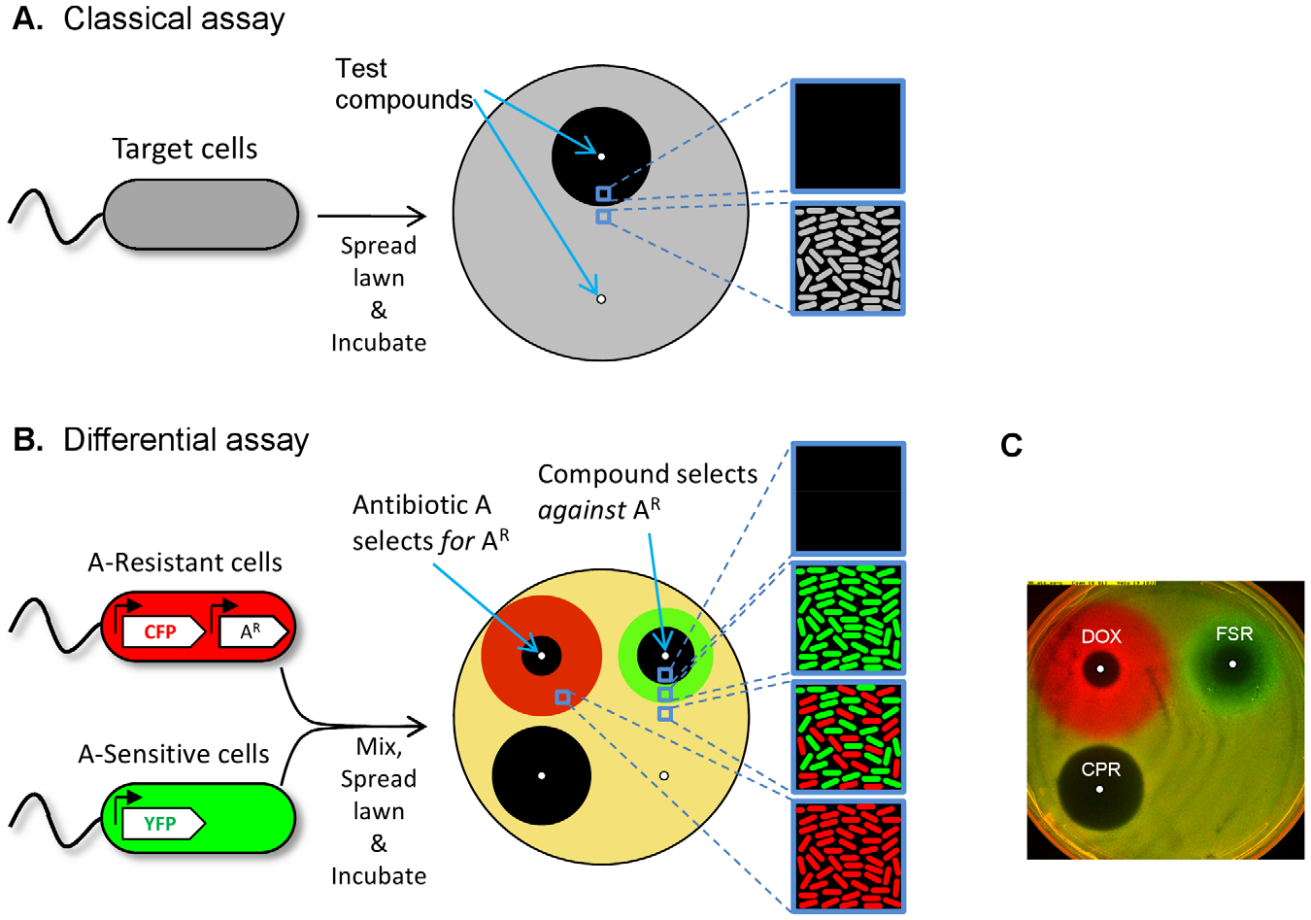
Competition-based agar diffusion assay for compounds that select against antibiotic resistance. **A**, A classical diffusion assay challenges a growing lawn of target bacterial cells (grey) with test compounds that diffuse from localized spots (white dots) on a nutrient agar plate. The size of the zones where bacterial growth is inhibited (dark region) reflects the potency of the compounds against the target strain. Non-inhibitory compounds or compounds to which the cells are resistant yield little or no zone of inhibition. Close-ups illustrate cellular growth across the plate. **B**, In the assay for differential selection, a pair of fluorescently-labeled strains, one drug-sensitive (YFP, shown in green) and one resistant (CFP, shown in red), are mixed in equal proportions, spread on nutrient agar and challenged with diffusing compounds. Fluorescent imaging yields ratios of the densities of the two strains across the plate. Non-selective conditions maintain the strains at 1:1 ratio (yellow), while compounds with different *relative* effect on the sensitive and resistant strains yield concentric zones of inhibition with different diameters resulting in a strongly dominant strain in the “zone of selection” between them (red or green rings). Selection by a drug for resistance to itself appears as a red ring, while a compound which selects against resistance is encircled by a green ring. Schematic close-ups illustrate cellular growth of the sensitive and resistant strains across the plate. **C**, Tetracycline-resistant (CFP-labeled strain t17cl, shown in red), and sensitive (YFP-labeled strain Wyl, shown in green) *E. coli* are mixed in equal proportions and spread over nutrient agar (Anhydrotetracycline in the agar induces expression of tetracycline resistance without inhibiting either strain; see Materials and Methods). Doxycycline generates strong selection for the tetracycline resistant strain (DOX, red ring), while fusaric acid selects for tetracycline sensitivity (FSR, green ring), and ciprofloxacin does not select, but inhibits the strains equally (CPR, no ring).

Our method is based on the agar diffusion assay for identifying the anti-microbial effect of compounds [10]. In this classical assay, a target microbial strain is uniformly seeded over the surface of a nutrient agar plate. Compounds to be tested are spotted locally on the agar surface. The plates are incubated and the cells grow as the test compounds diffuse outwards. Inhibitory compounds create zones of inhibition in the grown microbial lawn, where Minimal Inhibitory Concentrations (MICs) of the drugs were reached before the cells grew to detectable levels. The diameter of the inhibition zone reflects the susceptibility of the test organism (Figure 1A). The simple, inexpensive nature of the agar-diffusion assay, its wide dynamic range for effective drug concentrations, and compatibility with many organisms, has led to its extensive use in drug discovery and clinical diagnostics.

Building on this well-established technique, we developed a competition-based method for detecting the differential impact of compounds on the selective advantage of resistance. By competing two fluorescently labeled strains on the agar, we are able to observe not only absolute zones of inhibition, but also “zones of selection” in which compounds modulate the ratio between the strains (Figure 1B). Briefly, we mix drug-sensitive and drug-resistant strains, differentially labeled with constitutively-expressed Cyan and Yellow Fluorescent Protein (CFP, YFP) at 1∶1 ratio, and seed them onto nutrient agar plates (Figure 1B). We then spot test compounds onto the plates and incubate overnight. By imaging the grown-out plates for each fluorophore using a fluorescent plate imager (See Materials and Methods), we obtain the final ratios between the strains across the entire plate (Figure S1). Where the strains are not inhibited or are equally affected by a compound, the final ratio between the strains shows little change (yellow). However, compounds which differentially inhibit the sensitive and resistant strains generate inhibition zones with different diameters and thus a ring-shaped zone of selection between them where only one of the strains has grown (Figure 1B). While an antibiotic produces a ring of selection with the color of the strain resistant to it (Figure 1B, red ring), selection inverters, which give the sensitive strain an advantage, generate a ring of the opposite color (Figure 1B, green ring). Therefore, unlike the classical diffusion assay which reports on the *absolute* antimicrobial effect of a compound, the assay we developed is sensitive to the *differential* effect of compounds on competing microbial strains. We note though, that deviations from the truncation selection depicted in Figure 1 may complicate interpretation of the results and such analysis is beyond the scope of this paper.

We demonstrate our differential assay by focusing on resistance to tetracyclines, a generally safe, inexpensive, widely available, broad spectrum class of antibiotics whose overuse in both clinical and agricultural contexts has led to crippling levels of clinical resistance [11]. Tetracycline resistance is commonly due to the tetA-tetR operon, encoding the TetA efflux pump and the TetR repressor of pump expression in the absence of tetracyclines. We use a pair of *E. coli* strains with the same genetic background, with one sensitive to tetracyclines (labeled with YFP, shown in green), and the other carrying a chromosomally integrated cassette containing the *tetA-tetR* tetracycline resistance operon (labeled with CFP, shown in red; ‘dye swap’ controls using strains with the fluorescent labels reversed were also performed). Using these strains, our differential assay readily identified selection for tetracycline resistance by doxycycline (DOX, Figure 1C, red ring), and selection against resistance by fusaric acid (FSR, Figure 1C, green ring [6].

**Figure 2:**
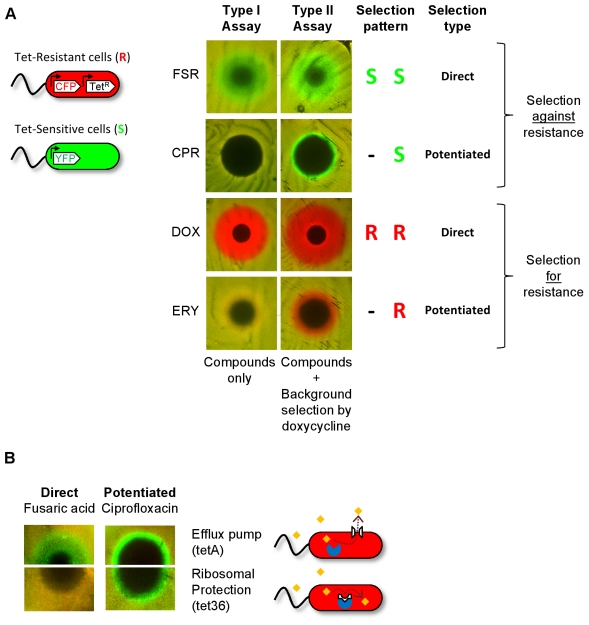
Assay discriminates two principal types of selection on antibiotic resistance: Direct and Potentiated. **A**, Tetracycline-resistant (tet^R^, CFP-labeled, strain t17cl, shown in red) and sensitive (tet^S^, YFP-labeled strain Wyl, shown in green) *E. coli* are mixed in equal proportions, seeded onto agar, and challenged with one of four compounds: fusaric acid (FSR), ciprofloxacin (CPR), doxycycline (DOX), erythromycin (ERY). The assay is performed either without (Type I Assay, Left), or with (Type II Assay, Right) background selection by doxycycline, which is added to the agar at a uniform concentration that imposes a slight (∼10%) growth inhibition of the sensitive strain (In Type I Assays, doxycycline is replaced by anhydrotetracycline at a uniform concentration that does not inhibit either strain, but assures expression of the tetA efflux pump). Selection patterns in Type I and II Assays classify compounds as selecting for or against tetracycline resistance (red, green) either directly (independently of background doxycycline selection, Assay I) or via potentiation (requiring background doxycycline selection, Assay II only). **B**, Direct and potentiated selection-inverters differ in their specificity with respect to the mechanism of resistance. This is illustrated by assay of selection inversion against a principal pair of mechanisms (White, in schematic) that protect ribosomes (Blue) from attack by tetracyclines (Yellow diamonds). Direct selection by fusaric acid is effective specifically against tetracycline resistance mediated by efflux pumps (tetA-bearing strain t17cl), but not against ribosomal protection (tet36-bearing strain GB(c)) in Type I Assays (Left). In contrast, ciprofloxacin exerts potentiated, general, selection against tetracycline resistance regardless of mechanism (Type II Assay, right).

The assay can be performed in the absence or in the presence of background selection by tetracycline, permitting identification and discrimination of compounds exerting direct or potentiated selection on resistance. In the absence of background selection by tetracycline (Type I Assay), the drug-sensitive and -resistant cells differ only in their expression of the resistance genes (induced by anhydrotetracycline in the agar at levels which do not affect growth; see Materials and Methods). Therefore, this assay is specific to ‘direct’ selection for and against resistance. Such direct selection is exhibited by doxycycline and fusaric acid, but not by ciprofloxacin or erythromycin (Figures 1C, 2A, “Type I Assay”). To reveal potentiated selection on tetracycline resistance, the same chemicals are assayed with a small quantity of doxycycline in the agar (generating ∼10% background growth inhibition of the sensitive relative to the resistant strain; Figure 2A, “Type II Assay”; see Materials and Methods). We find that the uniform background level of doxycycline potentiates selection by ciprofloxacin against tetracycline resistance (Figure 2A, Type II, CPR, green ring) and by erythromycin in favor of tetracycline resistance (Figure 2A, Type II, ERY, red ring). This observation is consistent with the suppressive versus synergistic interactions of doxycycline with ciprofloxacin and erythromycin, respectively. Since doxycycline suppresses the effect of ciprofloxacin [8], [12], its background presence decreases the radius of the ciprofloxacin inhibition zone of the tet^S^ strains. However, the tet^R^ strain, which pumps doxycycline out of the cell, does not enjoy this protection from ciprofloxacin and is thereby selected against. Conversely, since tetracycline and erythromycin interact synergistically, erythromycin potency is enhanced against the tet^S^, but not against the tet^R^ strain, resulting in relative selection for tetracycline resistance. This ‘Type II’ assay, in which the antibiotic to which the bacteria are resistant is added uniformly to the agar, therefore exposes potentiated, interaction-mediated, selection for and against resistance.

Together, the two assays comprise a simple means of identifying novel selection-inverting compounds and classifying them as exerting direct or potentiated selection on resistance (Figure 2A). An interesting prediction is that direct selection, which acts on specific resistance mechanism, will be exclusive to strains bearing this, but not other, resistance mechanism. On the other hand, potentiated selection on resistance, which occurs via selection on the consequences of reduced antibiotic effect generally conferred by resistance, should be insensitive to the particular resistance mechanism [8], [9].

To test this prediction and the generality of selection inversion with respect to mechanism of resistance, we applied our assay to another pair of strains, in which tetracycline resistance is mediated by a different mechanism. Specifically, we replaced the *tetA* tetracycline efflux pump with *tet(36)* which confers resistance to tetracyclines through a ribosomal protection mechanism [13]. We observe that tetracycline-potentiated selection inversion by ciprofloxacin indeed works on both of these very distinct resistance mechanisms, while direct selection inversion by fusaric acid is specific to the efflux-pump bearing microbes (Figure 2B).

Interestingly, the direct selection inverter fusaric acid is a natural product of microorganisms found on crops and in soil [14]. Other compounds produced by soil organisms are also known to reduce or invert the selective advantage of resistance [2], [4]. Since soil microbes are a rich source of antimicrobial compounds, the coexistence of resistant and sensitive species in the soil [15] may suggest a role for natural compounds which can act to invert selection for resistance. Motivated by these ideas, we set up our assay to search for soil species which produce chemical compounds that invert selection for antibiotic resistance.

**Figure 3:**
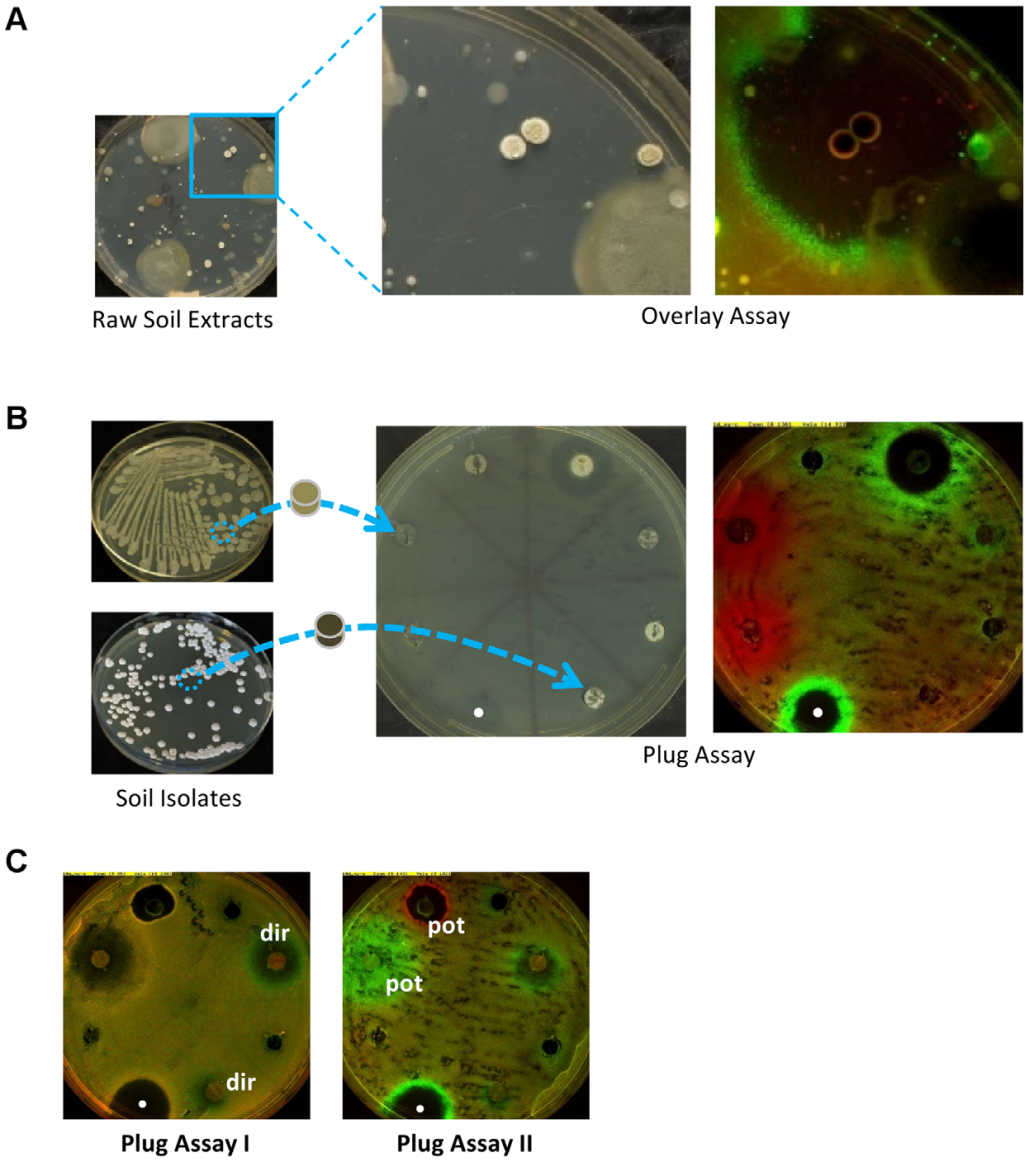
Screen of soil isolates for production of compounds that select against tetracycline resistance. Microbes extracted from soil are cultured on agar plates and then assayed for secretion of selection-inverters by (**A**) overlaying the mixed colony plates (left panel and zoom) with top agar containing tetracycline-resistant (red, CFP-labeled strain t17cl), and sensitive (green, YFP-labeled strain Wyl) *E.coli*, nutrients and a small quantity of doxycycline for background selection (Type II Assay, background selection). Fluorescent imaging (right panel) following incubation reveals colonies secreting compounds which diffused into the overlay leading to inhibition (clear zone) and selection inversion (green ring). Alternatively, (**B**) 5 mm agar plugs conditioned by purified soil isolates are transferred to Type II Assay plates spread with the *E.coli* test strains (Figure S2). Fluorescent imaging after incubation reveals selection inversion by secreted compounds in the plugs (green ring, top of plate in right panel). Pure ciprofloxacin is used as a plate control (white dot). **C**, Type I (left panel) and Type II (right panel) assays performed on pairs of conditioned agar plugs reveal whether selection inversion (green rings) by secreted compounds is direct (‘dir’, left panel), or potentiated (‘pot’, right panel). White dot indicates ciprofloxacin control.

We screened microbial isolates from diverse soil samples for production of compounds that invert selection for tetracycline resistance (Figure 3). Since soil microbes grow at varying rates and produce secondary metabolites at varying times, and because their isolation media is often different from our *E. coli* competition media, we decoupled the isolate growth and compound production phase of the assay from the resistant-sensitive competition phase. We first allow the isolates to grow and secrete compounds for up to two weeks under various culture conditions and only then apply our diffusion-based competition assay using two complementary methods: an “overlay assay” which is fast but crude, and a “plug assay” which is more elaborate, but less biased. In the “overlay assay”, we cover plates bearing mixed colonies of microbes extracted from soil samples with top agar containing a mixture of our labeled tetracycline-sensitive and resistant assay strains, fresh nutrients, and a small amount of doxycycline for background tetracycline selection. After incubation and imaging, rings of selection are found around colonies of faster growing soil microbes which have rapidly secreted compounds selecting on tetracycline resistance (Figure 3A). In the “plug assay”, we re-streak individual soil colonies on separate plates. Plugs of their conditioned agar are then transferred to holes in fresh assay plates spread with our test strain pair [16]. Similar to pure compounds spotted on the plates, secreted compounds diffuse from the plugs and some create zones of selection against resistance (Figure 3B, S2).

We have screened more than 1000 soil isolates for production of compounds that show differential selection on tetracycline resistance. Even this modest screen identified several isolates that excrete chemicals which bias selection in favor of sensitivity (6 isolates with stronger, and 16 with lesser effect). Interestingly, this number was not very different than the number of isolates with secretions that increase selection in favor of resistance (14 isolates with stronger and 8 with lesser effect). Furthermore, using our classification methodology (Figure 3C), we find that the majority of these compounds exhibit potentiated rather than direct selection on resistance (∼90% of the compounds show a zone of selection only in the presence of small amount of a tetracycline, Assay Type II). These results suggest that production of selection inverting compounds is common in soil microbes and that the presence of tetracyclines, even at sub-MIC levels can potentiate strong selective pressure, both for and against resistance, by other secreted compounds.

## Discussion

In summary, we developed a sensitive differential assay for compounds that select against antibiotic resistance. This assay is extendable to high-throughput screening (Figures S3, S4, Drawing S1, Methods S1), to resistance to other antibiotics and in other microbial species (Figure S5), and even to screen for drug specificity to pathogen versus human target isoforms (Figure S6, Methods S1) [17]. A similar chromogenic technique has also recently been used for sensitive biological detection of low levels of antibiotics and other toxins [18]. Applying our assay, we found that production of inverters of selection for tetracycline resistance appears to be relatively common amongst soil microbes, and that a majority of our hits appear to bias selection against tetracycline resistance only in the presence of some amount of a tetracycline. The combination of compounds that select for and against resistance may suggest further insights into the puzzling coexistence of antibiotic-resistant and sensitive species in the wild [15], [19], [20], [21].

## Materials and Methods

### Strains

See table of strains (Table S1).

### Assay Media

Assay plates consisted of M63 salts (2 g/l (NH_4_)_2_SO_4_, 13.6 g/l KH_2_PO_4_, 0.5 mg/l FeSO_4_ 7H_2_O), supplemented with 0.2% glucose, 0.01% casamino acids, 1 mM MgSO_4_, 1.5 µM thiamine, 1.5% bacto-agar (BD). Type I assay plates are further supplemented with 40 ng/ml anhydrotetracycline to induce *tetA* expression without affecting growth rate. Type II assay plates include ∼100 ng/ml doxycycline, which both induces *tetA* expression and confers ∼10% growth advantage to the resistant strain. Varying the doxycycline concentration roughly 2-fold affected the background ratios of the strains and the sensitivity of the measurement, but did not change the patterns of selection observed.

### Chemicals

Antibiotic stock solutions were prepared as follows: doxycycline hyclate (Sigma D-9891, 20 mg/ml in DMSO), erythromycin (Fluka 45673, 20 mg/ml in DMSO), fusaric Acid (Fluka 48205, 15 mg/ml in DMSO), ciprofloxacin (Fluka 17850, 0.015 mg/ml in 15 µM HCl), anhydrotetracycline (Acros AC23313, 0.2 mg/ml in EtOH).

### Assay for selection

Aliquot stocks of overnight stationary cultures (∼10^9^ cfu/ml) of assay strains, grown without antibiotic, are stored at −80°C. Stocks of a sensitive and resistant assay pair (one expressing CFP and one YFP) are thawed to room temperature, diluted 100-fold into PBS, and mixed. 100–200 µl of this mixture is then spread over the surface of each assay plate and allowed to dry. If assaying pure compounds, a 2–3 µl drop of each test compound is then spotted onto the agar surface and allowed to dry. The plates are then incubated at 30C for 20 hours, and then imaged for CFP and YFP signal.

### Fluorescent plate imager

Assay plates are imaged in CFP, YFP and white light channels using a custom, automated plate imager (Figure S3). Exciting illumination is provided by collimated light from filtered (Chroma, CFP:D436/20X, YFP:HQ500/20X) high intensity LEDs (www.luxeonstar.com, CFP: Royal Blue lambertian Luxeon V star, YFP: Cyan lambertian Luxeon V star). Emitted light is filtered (Chroma, CFP:D480/30 m, YFP:HQ535/30 m) and captured by a digital camera (Olympus SP-350, Cam2Com). Custom software (AutoIt) commands image acquisition, and switches lamps and filters using an automated filter wheel (Thorlabs FW102B) mated to a modified rotary switch (Allied Electronics). Images are automatically analyzed using custom software (MATLAB).

### Image analysis

Images of the plates in each fluorescence channel are divided by images of uniform fluorescent sheets to correct for fluorescence shading artifacts. Image regions (50×50 pixel) representing areas of no growth (black region) and test compound-free background growth (yellow region) are manually specified. The intensity data for each fluorescent channel are then linearly rescaled to give ∼1% and 55% signal saturation at the specified yellow and black regions respectively. These normalized images are displayed as the red (resistant strain) and green (sensitive strain) channels of a false color image.

### Soil isolation

Cells were extracted from soil samples by vortexing ∼1 g soil with 5 ml PBS, plating dilutions on a variety of solid media (Potato Dextrose Agar, Yeast Malt Agar (ISP-2), Inorganic Salts Starch Agar (ISP-4), Bennett's Agar, Luria-Bertani Agar (LB), Dilute Nutrient Agar (DNB)), and incubating for 1–2 weeks at room temperature. To complete isolation for plug-based assay or storage, individual colonies were picked from these extract plates, and re-struck in isolation. Strains were stored in PBS containing 25% glycerol, mixing, and freezing at −80°C.

### Overlay Assay

The ‘Overlay’ assay operates on the principle that gradients of excreted selective compounds are formed around colonies of their producers, and that such compounds diffuse as gradients into thin layers of agar containing assay strains which are laid over them. The assay is performed on 1–2 week-old soil extract plates typically bearing 15–50 medium-to-large colonies of different strains. 5 ml of molten Assay Top Agar (identical to assay agar, but containing 0.75% agar, 0.4% glucose, 0.02% casamino acids and 300 ng/ml doxycycline hyclate) at 40°C is mixed with a 50 µl thawed aliquot (∼5×10^7^ cells per plate) of each strain of an assay pair, poured uniformly over the surface of a pre-warmed isolation plate, and allowed to solidify. The plate is then incubated for 20 hours at 30°C prior to imaging. Image analysis is performed as described above, and soil extract colonies with distinct zones of selection around them (green rings) are picked through the overlay and re-struck to isolation.

### Plug Assay

5 mm plugs of agar, conditioned by individual isolates for 1–2 weeks, are removed from the plates and replaced into 5 mm holes punched in standard assay plates spread with assay strain pairs. The plates are incubated for 20 hours at 30°C, imaged and analyzed as described above. Plugs from plates conditioned by control strains known to produce tetracyclines or fusaric acid yield results similar to the pure compounds (Figure S2).

### Screen for microbial producers of selection modulating compounds

Plug and overlay assays were applied to screen for natural isolates that modify selection of tetracycline resistance using a Type II assay (∼700 by plug assay; ∼60 plates of about 50 colonies each using the overlay assay). We identified and isolated 30 colonies showing clear zone of selections either for or against resistance. Using Type I and Type II plug assays, compounds produced by the isolates were identified as selecting either directly or via potentiated selection on tetracycline resistance (Figure 2).

## Supporting Information

Figure S1 **Cell-ratio measurements from two-color fluorescence imaging agree quantitatively with ratios of labeled** ***E.coli*** **in spot gradients.** CFP and YFP-labeled *E. coli*, mixed in different ratios, were inoculated onto M63 Glucose minimal agar (∼10^5^ cells/spot), grown overnight, and imaged (Merged fluorescent images, right panels). The images were corrected for shading, and fluorescence intensities collected over 80X80 pixel regions within the mixed colonies (Red squares, right panels). Using a linear fluorescence/cell assumption and average signals from the selected regions and the background, we calculate background autofluorescence, CFP/YFP signal crosstalk, and the fraction of YFP-labeled cells per spot. Calculated fractions of YFP-labeled cells reproducibly reflect the inoculated fractions.
(TIF)

Figure S2 **Agar plugs conditioned by microbial producers of selection-modulating compounds give rise to similar zones of selection as pure compounds.** (**A**) An agar plug (Yeast Malt media) conditioned by *Streptomyces rimosus* (Oxytetracycline producer) generates a zone of selection for tetracycline resistance similar to the effect of pure doxycyline (red rings). (**B**) An agar plug (Potato Dextrose media) conditioned by *Giberella fujikuroi* (fusaric acid producer) generates a zone of selection against tetracycline resistance, similar to the effect of pure fusaric Acid (green rings). Type II assays using tetracycline resistant (YFP, red, tetA efflux pump) and sensitive (CFP, green) *E.coli*.
(TIF)

Figure S3 **The assay for selection is readily adapted to a high-throughput screening format.** (**A,B**) Linear diffusion along channel-wells in a custom plate (**B**) increases the spatial density of the assay compared to circular diffusion (**A**). Zones of selection for/against resistance appear as red/green bands rather than circles (compare blue frames). (**C**) Automated image analysis yields final ratios between sensitive and resistant strains along each well. The corresponding compounds are thus classified as selecting for resistance (red traces), against resistance (green traces), or as neutral (yellow traces). (**D**) Custom multicolor fluorescence imager. (**E**) Custom 2x24 channel-well assay plates allow integration with standard 384-well format chemical libraries.
(TIF)

Figure S4 **2x24 channel-well linear diffusion plate, in standard microplate dimensions.** For CAD specifications, see Drawing S1.
(TIF)

Figure S5 **Strains for assaying selection on resistance to four different antibiotics.** Fluorescent plate images of different fluorescently-labeled sensitive (green, YFP-labeled) and antibiotic-resistant (red, CFP-labeled) assay strains in the presence of four antibiotics. Strains are differentially resistant to chloramphenicol (Chl), erythromycin (Ery), neomycin (Neo), and tetracycline (Tet), and comprise both Gram-negative (*E.coli*) and Gram-positive (*B.subtilis*) bacteria. Red rings along the main diagonal show classical selection for resistance. Dye swaps yield identical results (not shown).
(TIF)

Figure S6 **The assay for selection provides an efficient means of identifying compounds that select for human over** ***plasmodium*** **dihydrofolate reductase.** Infection chemotherapeutics requires windows of drug concentration where molecular targets in the pathogen are preferentially inhibited while patient isoforms are left unmolested. By assaying for relative selection using fluorescently-labeled *E.coli* which are dependent on dihydrofolate reductase (DHFR) of either human (YFP-labeled strain Hky, green) or *plasmodium falciparum* (CFP-labeled strain P(0)c, red) origin [1], [2], [3], we sensitively and directly identify compounds which preferentially inhibit the pathogen target isoform. While a non-discriminatory drug (kanamycin, KAN) inhibits both strains equally, a known anti-malarial DHFR inhibitor (pyrimethamine, PYR) creates a zone of selection, indicating a range of concentrations of strong selection for the human over plasmodium-derived DHFR (Green ring). Native bacterial DHFR activity is chemically removed by 1 µg/ml Trimethoprim in the agar [3]. 1.→Djapa LY, Basco LK, Zelikson R, Rosowsky A, Djaman JA, et al. (2007) Antifolate screening using yeast expressing Plasmodium vivax dihydrofolate reductase and in vitro drug susceptibility assay for Plasmodium falciparum. Molecular and Biochemical Parasitology 156: 89-92. 2.→Gerum AB, Ulmer JE, Jacobus DP, Jensen NP, Sherman DR, et al. (2002) Novel Saccharomyces cerevisiae screen identifies WR99210 analogues that inhibit Mycobacterium tuberculosis dihydrofolate reductase. Antimicrobial Agents and Chemotherapy 46: 3362-3369. 3.→Lozovsky ER, Chookajorn T, Brown KM, Imwong M, Shaw PJ, et al. (2009) Stepwise acquisition of pyrimethamine resistance in the malaria parasite. Proc Natl Acad Sci U S A 106: 12025-12030.
(TIF)

Table S1 **List of strains and plasmids.**
(PDF)

Methods S1 **Supplementary methods for high-throughput screening using 2x24 channel-well plates and screening for differential inhibition of human and plasmodium dihydrofolate reductase.**
(PDF)

Drawing S1 **Technical CAD drawing (.dxf) of 2×24 channel-well linear diffusion plates.**
(DXF)

## References

[1] Taubes G (2008). The bacteria fight back.. Science.

[2] Coleman K, Athalye M, Clancey A, Davison M, Payne DJ (1994). Bacterial-Resistance Mechanisms as Therapeutic Targets.. Journal of Antimicrobial Chemotherapy.

[3] Li XZ, Nikaido H (2009). Efflux-Mediated Drug Resistance in Bacteria An Update.. Drugs.

[4] Palmer AC, Angelino E, Kishony R (2010). Chemical decay of an antibiotic inverts selection for resistance.. Nature Chemical Biology.

[5] Li Q, Lee JY, Castillo R, Hixon MS, Pujol C (2002). NB2001, a novel antibacterial agent with broad-spectrum activity and enhanced potency against beta-lactamase-producing strains.. Antimicrobial Agents and Chemotherapy.

[6] Bochner BR, Huang HC, Schieven GL, Ames BN (1980). Positive Selection for Loss of Tetracycline Resistance.. Journal of Bacteriology.

[7] Szybalski W, Bryson V (1952). Genetic Studies on Microbial Cross Resistance to Toxic Agents.1. Cross Resistance of Escherichia-Coli to 15 Antibiotics.. Journal of Bacteriology.

[8] Chait R, Craney A, Kishony R (2007). Antibiotic interactions that select against resistance.. Nature.

[9] Bollenbach T, Quan S, Chait R, Kishony R (2009). Nonoptimal Microbial Response to Antibiotics Underlies Suppressive Drug Interactions.. Cell.

[10] Lorian V (2005). Antibiotics in laboratory medicine..

[11] Chopra I, Roberts M (2001). Tetracycline antibiotics: Mode of action, applications, molecular biology, and epidemiology of bacterial resistance.. Microbiology and Molecular Biology Reviews.

[12] Yeh P, Tschumi AI, Kishony R (2006). Functional classification of drugs by properties of their pairwise interactions.. Nature Genetics.

[13] Whittle G, Whitehead TR, Hamburger N, Shoemaker NB, Cotta MA (2003). Identification of a new ribosomal protection type of tetracycline resistance gene, tet(36), from swine manure pits.. Applied and Environmental Microbiology.

[14] Bacon CW, Porter JK, Norred WP, Leslie JF (1996). Production of fusaric acid by Fusarium species.. Applied and Environmental Microbiology.

[15] D'Costa VM, McGrann KM, Hughes DW, Wright GD (2006). Sampling the antibiotic resistome.. Science.

[16] Campbell AH (1960). The Search for New Antibiotics.. British Medical Bulletin.

[17] Djapa LY, Basco LK, Zelikson R, Rosowsky A, Djaman JA (2007). Antifolate screening using yeast expressing Plasmodium vivax dihydrofolate reductase and in vitro drug susceptibility assay for Plasmodium falciparum.. Molecular and Biochemical Parasitology.

[18] Liu A, Fong  A, Becket E, Yuan J, Tamae C (2010). Selective Advantage of Resistant Strains at Trace Levels of Antibiotics: A Simple and Ultrasensitive Color Test for the Detection of Antibiotics and Genotoxic Agents.. Antimicrobial Agents and Chemotherapy.

[19] Waksman SA (1961). The Role of Antibiotics in Nature.. Perspectives in Biology and Medicine.

[20] Martinez JL (2008). Antibiotics and antibiotic resistance genes in natural environments.. Science.

[21] Allen HK, Donato J, Wang HH, Cloud-Hansen KA, Davies J (2010). Call of the wild: antibiotic resistance genes in natural environments.. Nature Reviews Microbiology.

