# Supplementary figures and images for: A Differential Drug Screen for Compounds That Select Against Antibiotic Resistance
Source: PLoS One. 2010 Dec 8;5(12):e15179. doi: 10.1371/journal.pone.0015179 (PMC2999542; doi:10.1371/journal.pone.0015179)

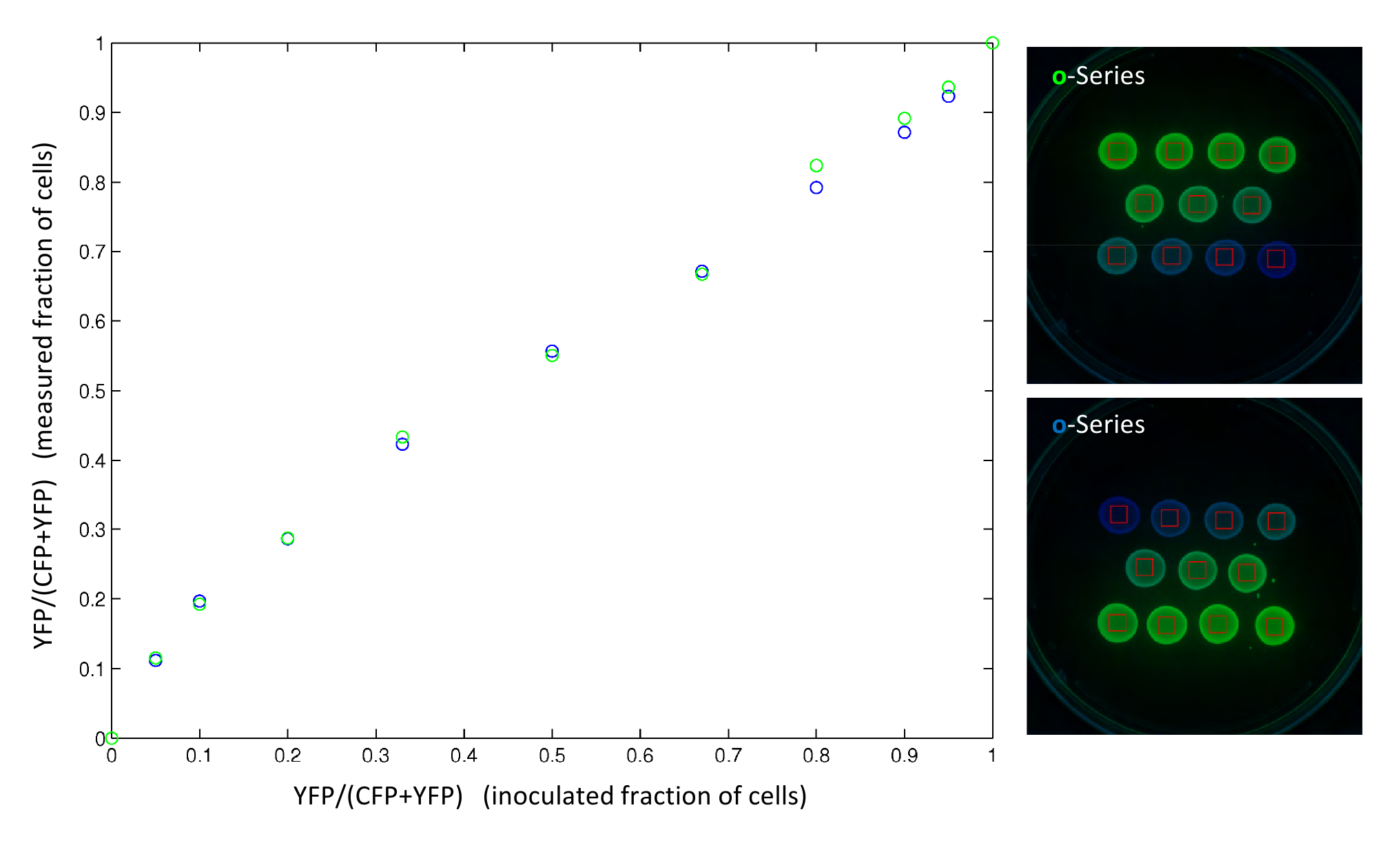

Supplement: Figure S1 — Cell-ratio measurements from two-color fluorescence imaging agree quantitatively with ratios of labeled E.coli in spot gradients. CFP and YFP-labeled E. coli, mixed in different ratios, were inoculated onto M63 Glucose minimal agar (∼105 cells/spot), grown overnight, and imaged (Merged fluorescent images, right panels). The images were corrected for shading, and fluorescence intensities collected over 80X80 pixel regions within the mixed colonies (Red squares, right panels). Using a linear fluorescence/cell assumption and average signals from the selected regions and the background, we calculate background autofluorescence, CFP/YFP signal crosstalk, and the fraction of YFP-labeled cells per spot. Calculated fractions of YFP-labeled cells reproducibly reflect the inoculated fractions. (TIF) [file pone.0015179.s001.tif]

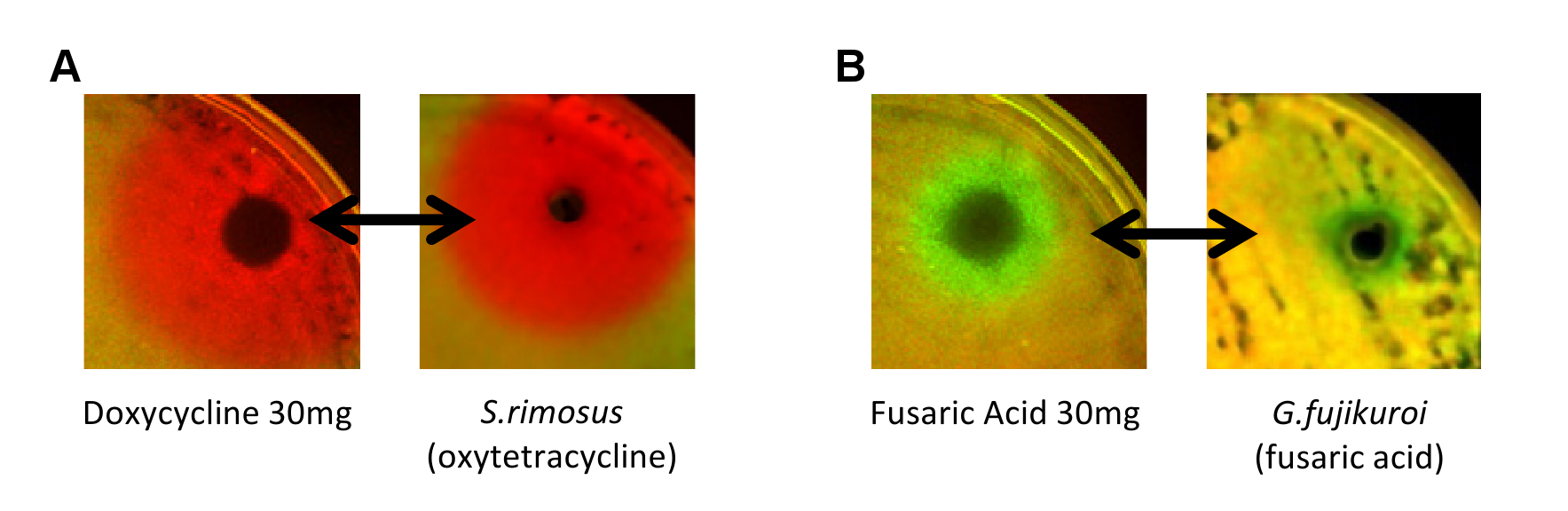

Supplement: Figure S2 — Agar plugs conditioned by microbial producers of selection-modulating compounds give rise to similar zones of selection as pure compounds. (A) An agar plug (Yeast Malt media) conditioned by Streptomyces rimosus (Oxytetracycline producer) generates a zone of selection for tetracycline resistance similar to the effect of pure doxycyline (red rings). (B) An agar plug (Potato Dextrose media) conditioned by Giberella fujikuroi (fusaric acid producer) generates a zone of selection against tetracycline resistance, similar to the effect of pure fusaric Acid (green rings). Type II assays using tetracycline resistant (YFP, red, tetA efflux pump) and sensitive (CFP, green) E.coli. (TIF) [file pone.0015179.s002.tif]

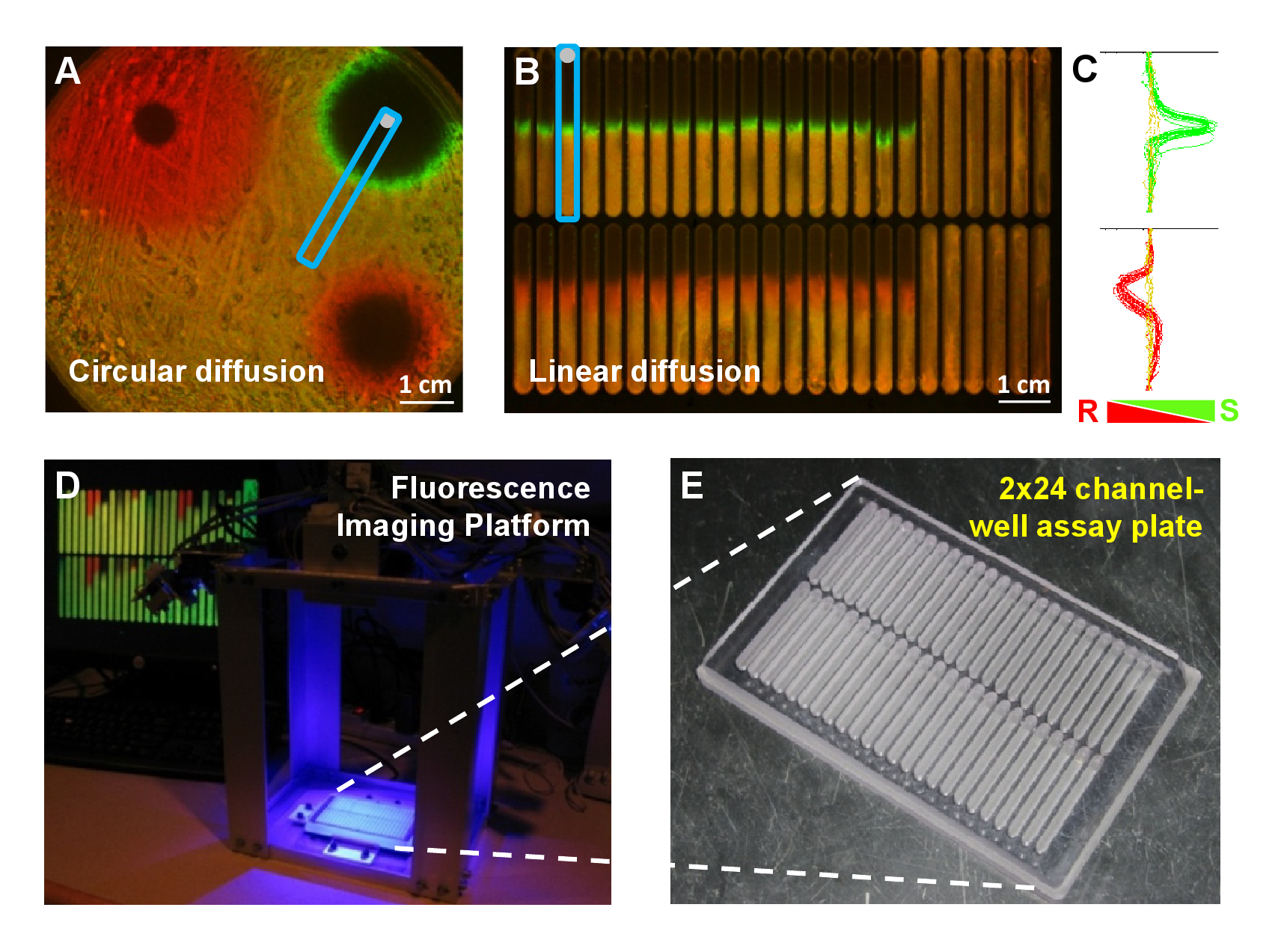

Supplement: Figure S3 — The assay for selection is readily adapted to a high-throughput screening format. (A,B) Linear diffusion along channel-wells in a custom plate (B) increases the spatial density of the assay compared to circular diffusion (A). Zones of selection for/against resistance appear as red/green bands rather than circles (compare blue frames). (C) Automated image analysis yields final ratios between sensitive and resistant strains along each well. The corresponding compounds are thus classified as selecting for resistance (red traces), against resistance (green traces), or as neutral (yellow traces). (D) Custom multicolor fluorescence imager. (E) Custom 2x24 channel-well assay plates allow integration with standard 384-well format chemical libraries. (TIF) [file pone.0015179.s003.tif]

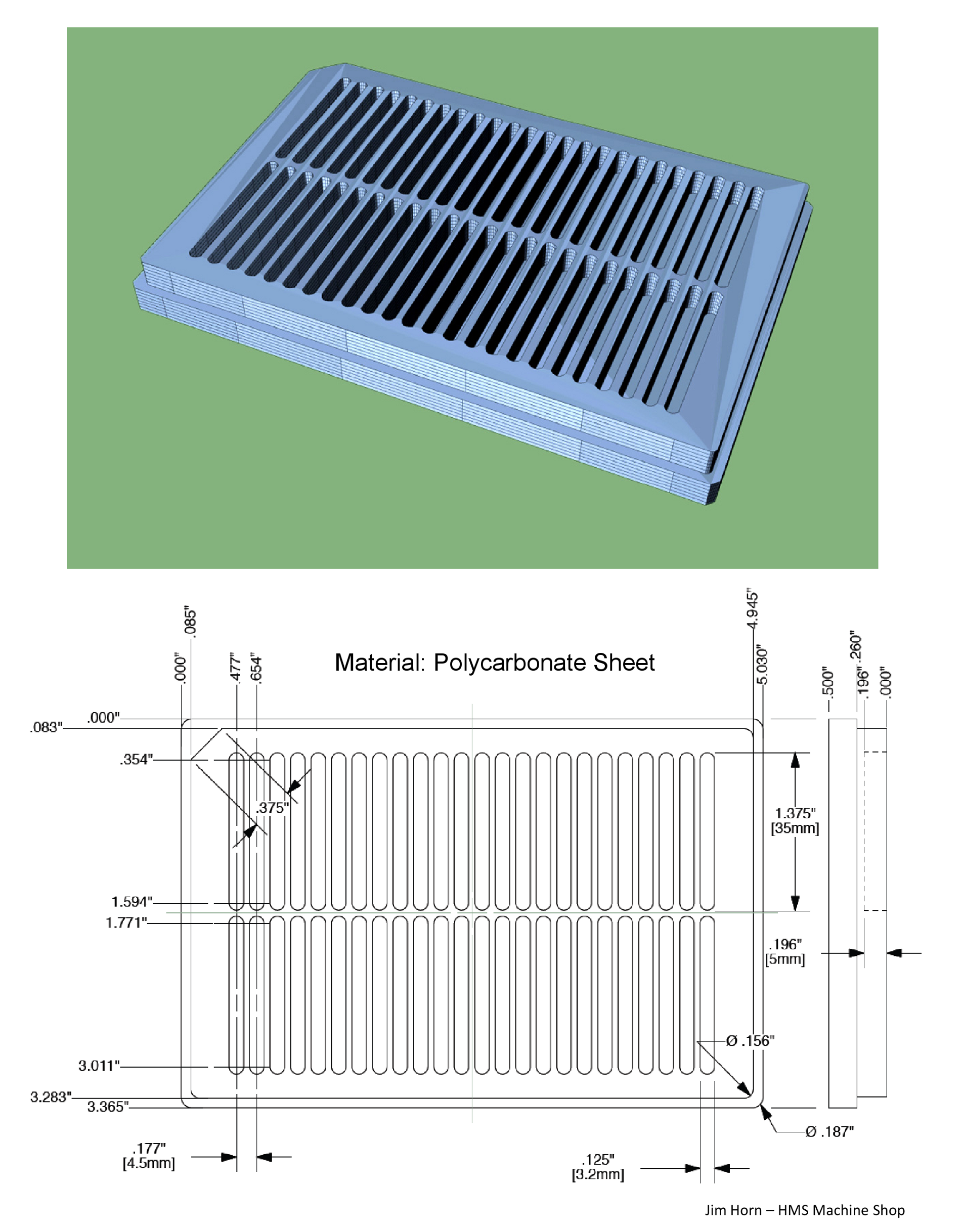

Supplement: Figure S4 — 2x24 channel-well linear diffusion plate, in standard microplate dimensions. For CAD specifications, see Drawing S1. (TIF) [file pone.0015179.s004.tif]

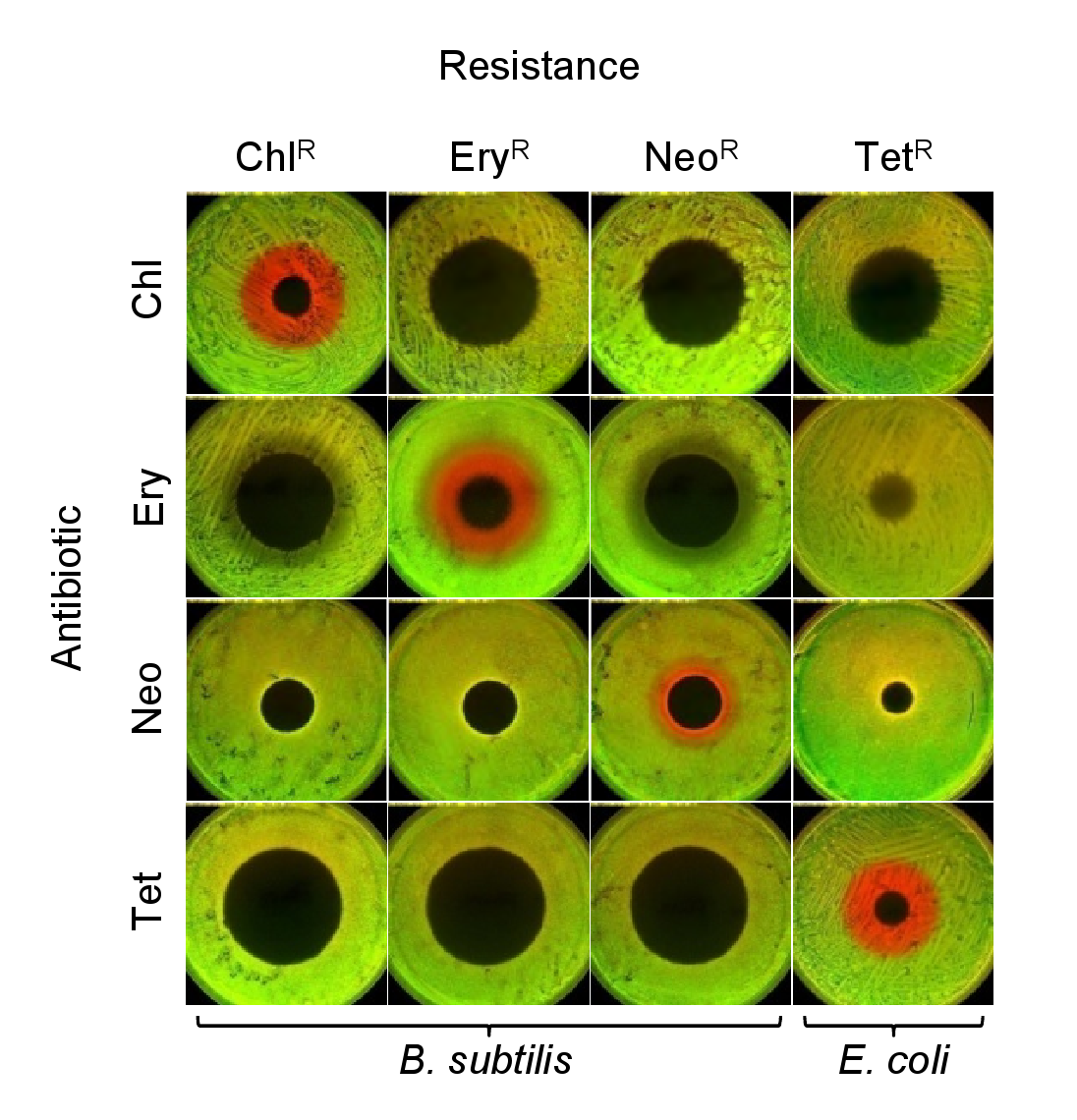

Supplement: Figure S5 — Strains for assaying selection on resistance to four different antibiotics. Fluorescent plate images of different fluorescently-labeled sensitive (green, YFP-labeled) and antibiotic-resistant (red, CFP-labeled) assay strains in the presence of four antibiotics. Strains are differentially resistant to chloramphenicol (Chl), erythromycin (Ery), neomycin (Neo), and tetracycline (Tet), and comprise both Gram-negative (E.coli) and Gram-positive (B.subtilis) bacteria. Red rings along the main diagonal show classical selection for resistance. Dye swaps yield identical results (not shown). (TIF) [file pone.0015179.s005.tif]

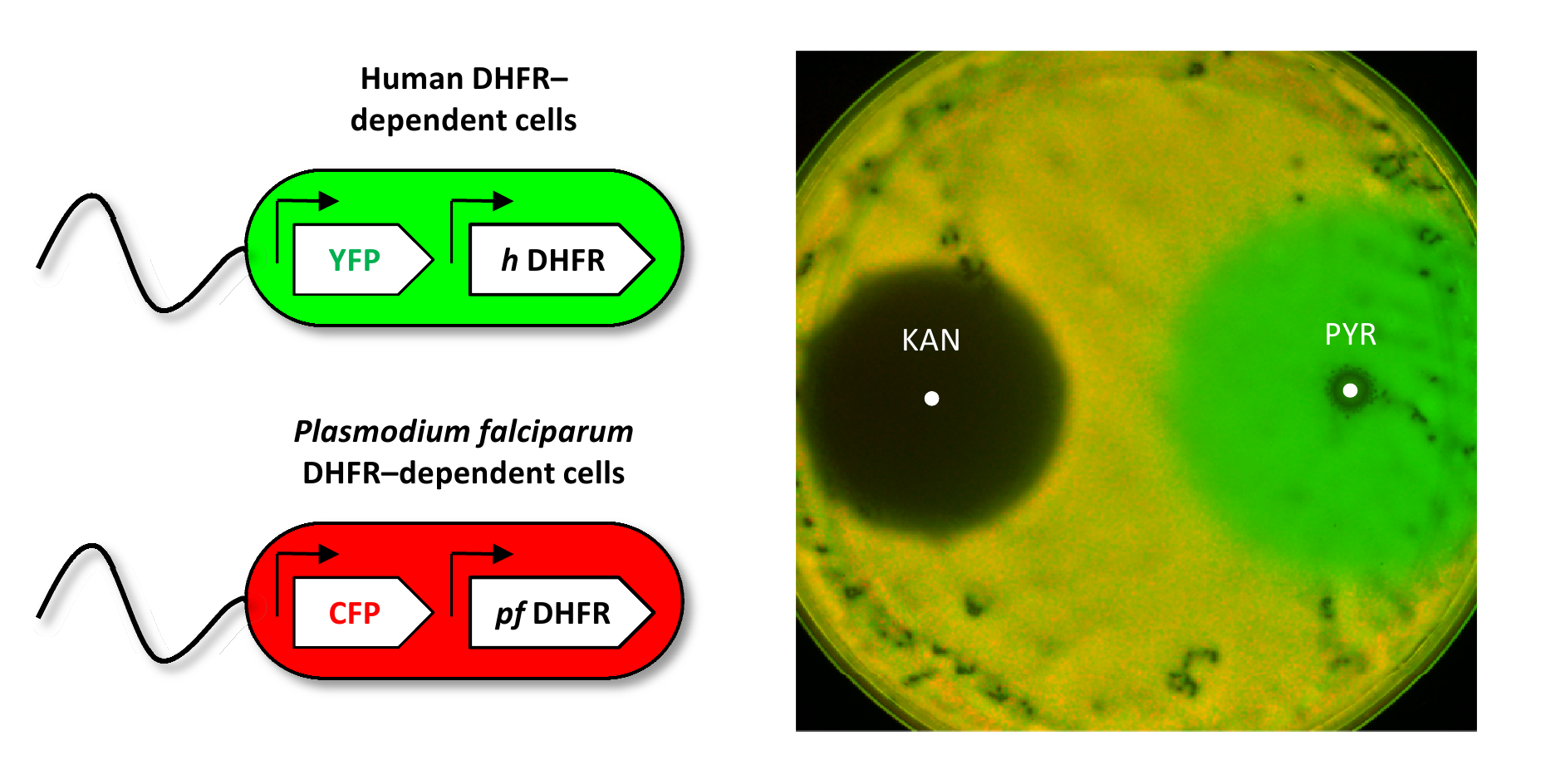

Supplement: Figure S6 — The assay for selection provides an efficient means of identifying compounds that select for human over plasmodium dihydrofolate reductase. Infection chemotherapeutics requires windows of drug concentration where molecular targets in the pathogen are preferentially inhibited while patient isoforms are left unmolested. By assaying for relative selection using fluorescently-labeled E.coli which are dependent on dihydrofolate reductase (DHFR) of either human (YFP-labeled strain Hky, green) or plasmodium falciparum (CFP-labeled strain P(0)c, red) origin [1], [2], [3], we sensitively and directly identify compounds which preferentially inhibit the pathogen target isoform. While a non-discriminatory drug (kanamycin, KAN) inhibits both strains equally, a known anti-malarial DHFR inhibitor (pyrimethamine, PYR) creates a zone of selection, indicating a range of concentrations of strong selection for the human over plasmodium-derived DHFR (Green ring). Native bacterial DHFR activity is chemically removed by 1 µg/ml Trimethoprim in the agar [3]. 1.→Djapa LY, Basco LK, Zelikson R, Rosowsky A, Djaman JA, et al. (2007) Antifolate screening using yeast expressing Plasmodium vivax dihydrofolate reductase and in vitro drug susceptibility assay for Plasmodium falciparum. Molecular and Biochemical Parasitology 156: 89-92. 2.→Gerum AB, Ulmer JE, Jacobus DP, Jensen NP, Sherman DR, et al. (2002) Novel Saccharomyces cerevisiae screen identifies WR99210 analogues that inhibit Mycobacterium tuberculosis dihydrofolate reductase. Antimicrobial Agents and Chemotherapy 46: 3362-3369. 3.→Lozovsky ER, Chookajorn T, Brown KM, Imwong M, Shaw PJ, et al. (2009) Stepwise acquisition of pyrimethamine resistance in the malaria parasite. Proc Natl Acad Sci U S A 106: 12025-12030. (TIF) [file pone.0015179.s006.tif]
