# Supplementary material for: A Differential Drug Screen for Compounds That Select Against Antibiotic Resistance
Source: PLoS One. 2010 Dec 8;5(12):e15179. doi: 10.1371/journal.pone.0015179 (PMC2999542; doi:10.1371/journal.pone.0015179)
Supplement: Table S1 — List of strains and plasmids. (PDF) [file pone.0015179.s007.pdf]

Table S1

Strain or Plasmid	Type	Notes	Source
Wyl	MC4100-YFP / pCS-λ	*E.coli* assay strain, Tet^S	[1]
Wcl	MC4100-CFP / pCS-λ	*E.coli* assay strain, Tet^S	[1]
t17yl	Wyl, ycaD-ycaM::Tn10	*E.coli* Tet^R(efflux)	[1]
t17cl	Wcl, ycaD-ycaM::Tn10	*E.coli* Tet^R(efflux)	[1]
pGW155B	pGEM-T::tet(36)	*E.coli* Tet^R(ribosomal protection)	[2]
GB(y)	MC4100-YFP / pGW155B	*E.coli* Tet^R(ribosomal protection)	This paper
GB(c)	MC4100-CFP / pGW155B	*E.coli* Tet^R(ribosomal protection)	This paper
NRRL 2234	*Streptomyces rimosus*	Oxytetracycline production	USDA-ARS
ATCC 14164	*Giberella fujikuroi*	Fusaric acid production	ATCC
PY79	*B. subtilis* wild-type strain		Dr. Michael Elowitz
pEr::Nm	Ery^R::Neo^R	Neo^R	BGSC, [3]
pEr::Pm	Ery^R::Phm^R	Phm^R	BGSC, [3]
pEr::Cm	Ery^R::Chl^R	Chl^R	BGSC, [3]
pCm::Er	Chl^R::Ery^R	Ery^R	BGSC, [3]
pSpE2X	Vector for insertion into *E. faecalis*-derived Sp^R		[4], This paper
NmEY	PY79, AmyE::(YFP,Neo^R)	*B. subtilis* assay strain, Chl^S	This paper
1AC	PY79, AmyE::(CFP,Neo^R,Chl^R)	*B. subtilis* assay strain, Chl^R	This paper
PmEY	PY79, AmyE::(YFP,Phm^R)	*B. subtilis* assay strain, Neo^S	This paper
7AC	PY79, AmyE::(CFP,Phm^R,Neo^R)	*B. subtilis* assay strain, Neo^R	This paper
NmNY	PY79, AmyE::(YFP,Neo^R)	*B. subtilis* assay strain, Ery^S	This paper
4AC	PY79, AmyE::(CFP,Neo^R,Ery^R)	*B. subtilis* assay strain, Ery^R	This paper
pZA31	p15A ori, Chl^R, P_{Ltet}		[5]
pET-17b	Expression vector		Novagen
pDONR221::hDHFR	human DHFR cDNA	GenBank: DQ892816.2	HIP/PlasmID
DHFR(0000)	HMS174(DE3)/pET-17b::pfDHFR(0000)	*E.coli* expressing *P. falciparum* DHFR	[6]
Hky	HMS174(DE3)/pET-17b::hDHFR, pZA31::YFP	human DHFR assay strain	This paper
P(0)c	DHFR(0000) / pZA31::CFP	*P. falciparum* DHFR assay strain	This paper

1. Chait R, Craney A, Kishony R (2007) Antibiotic interactions that select against resistance. Nature 446: 668-671.
2. Whittle G, Whitehead TR, Hamburger N, Shoemaker NB, Cotta MA, et al. (2003) Identification of a new ribosomal protection type of tetracycline resistance gene, tet(36), from swine manure pits. Applied and Environmental Microbiology 69: 4151-4158.
3. Steinmetz M, Richter R (1994) Plasmids Designed to Alter the Antibiotic-Resistance Expressed by Insertion Mutations in Bacillus-Subtilis, through in-Vivo Recombination. Gene 142: 79-83.
4. Leblanc DJ, Lee LN, Inamine JM (1991) Cloning and Nucleotide Base Sequence-Analysis of a Spectinomycin Adenyltransferase Aad(9) Determinant from Enterococcus-Faecalis. Antimicrobial Agents and Chemotherapy 35: 1804-1810.
5. Lutz R, Bujard H (1997) Independent and tight regulation of transcriptional units in Escherichia coli via the LacR/O, the TetR/O and AraC/I-1-I-2 regulatory elements. Nucleic Acids Research 25: 1203-1210.
6. Lozovsky ER, Chookajorn T, Brown KM, Imwong M, Shaw PJ, et al. (2009) Stepwise acquisition of pyrimethamine resistance in the malaria parasite. Proc Natl Acad Sci U S A 106: 12025-12030.
