# Supplementary material for: A Differential Drug Screen for Compounds That Select Against Antibiotic Resistance
Source: PLoS One. 2010 Dec 8;5(12):e15179. doi: 10.1371/journal.pone.0015179 (PMC2999542; doi:10.1371/journal.pone.0015179)
Supplement: Methods S1 — Supplementary methods for high-throughput screening using 2x24 channel-well plates and screening for differential inhibition of human and plasmodium dihydrofolate reductase. (PDF) [file pone.0015179.s008.pdf]

Methods S1

High-throughput screening using 2x24 well plates. 2x24 channel-well assay plates in microplate format permit higher density screening, and automated integration with 384-well chemical libraries and image analysis (Figures S3,S4). The plates described in Figure S4 and Drawing S1 are prepared by loading each well with 0.5 ml of assay agar. The plates are then inverted and dipped into a 100-fold dilution of thawed assay strain stock mixture in PBS, righted, and allowed to dry. Test compounds are transferred to ends of the agar strips in ~1ul volumes, via pipette or pin tool, at positions corresponding to rows A and I of a 384-well plate. The plates are incubated for 20 hours at 30 ^{o}C, imaged, and the standard analysis performed. Ratios between the strains along each well are extracted and automatically scanned for deviations from neutral selection using custom MATLAB software.

Screen for chemical inhibitors which preferentially target pathogen isoforms of dihydrofolate reductase. The assay methodology can be used to rapidly screen for compounds which preferentially inhibit *plasmodium falciparum* dihydrofolate reductase (DHFR) over human-derived DHFR (Figure S6). YFP and CFP-labeled *E.coli* that constitutively express either human (strain HKy) or *plasmodium falciparum*-derived (strain P(0)c) DHFR respectively, form an assay pair. In the presence of ~1 µg/ml trimethoprim, the native bacterial DHFR is completely inhibited and the cells depend entirely upon the exogenous DHFR for essential pyrimidine synthesis[1]. LB agar plates containing 1 µg/ml trimethoprim are spread with 100-200 µl of 1:1 thawed aliquots of assay strains diluted 10-fold in PBS. The plates are dried and 2-3 µl of the test compounds are spotted onto the agar. The plates are then incubated for 20 hours at 30 ^{o}C, imaged and analyzed for rings of differential selection against the plasmodium-derived DHFR.

1. Lozovsky ER, Chookajorn T, Brown KM, Imwong M, Shaw PJ, et al. (2009) Stepwise acquisition of pyrimethamine resistance in the malaria parasite. Proc Natl Acad Sci U S A 106: 12025-12030.
